# New Appliances and Things Medical

**Published:** 1900-07-14

**Authors:** 


### NEW APPLIANCES AND THINGS MEDICAL.

[We shall be glad to receive, at our Office, 28 & 29, Southampton Street, Strand, London, W.C., from the manufacturers, specimens of all new preparations and appliances which may be brought out from time to time.]

### CHAMPAGNE FOR MEDICAL PURPOSES.

(LITTKE'S CHAMPAGNE: INGRAM AND ROYLE, LTD., 26, UPPER THAMES STREET, LONDON, E.C.)

WE have received two samples of this champagne; one a very dry wine, and the other possessing slight saccharine properties. We have examined both these wines, which are undoubtedly of a high class, and find them to be pure and wholesome varieties well suited to invalid purposes.

### IMPROVED INSTEP ARCH SOCK.

(THOMAS HOLLAND, 40, SOUTH AUDLEY STREET, LONDON, W.)

THIS new instep arch appears to possess advantages over most of the older patterns with which we are acquainted. The modified valgus pad which is built up into the sock is carefully moulded so as to supply an even and well-distributed pressure on the inside of the arch and the calcaneo-scaphoid ligament. There are no sharp edges to chafe the tender and often inflamed ligamentous tissue associated with flat foot. Those who are victims of this most intractable condition will do well to give this new arch support a trial.

